# Learnings from providing integrated health, housing and wider care for people rough sleeping during the COVID- 19 pandemic: a national qualitative study of the ‘Everyone In’ policy initiative

**DOI:** 10.1186/s12913-025-12713-w

**Published:** 2025-04-15

**Authors:** Neha Jain, Emma A. Adams, Kate Haddow, Jo Brown, Dan Bleksley, Stephan Morrison, Joanna Kesten, Kelly Howells, Caroline Sanders, Ashley J. Adamson, Eileen Kaner, Sheena E. Ramsay

**Affiliations:** 1 Population Health Sciences Institute, Newcastle University, Baddiley Clark Building, Richardson Road, Newcastle Upon Tyne, NE2 4 AX UK; 2 Groundswell, London, UK; 3 Population Health Sciences, Bristol Medical School, University of Bristol, Bristol, UK; 4 The National Institute for Health and Care Research Applied Research Collaboration West (NIHR ARC West), University Hospitals Bristol and Weston NHS Foundation Trust, Bristol, UK; 5 The National Institute for Health and Care Research (NIHR) Health Protection Research Unit (HPRU) in Behavioural Science and Evaluation, University of Bristol, Bristol, UK; 6 Division of Population Health, Health Services Research and Primary Care, University of Manchester, Manchester, UK; 7 The National Institute for Health Research (NIHR) Greater Manchester Patient Safety Research Collaboration, Manchester, UK

**Keywords:** Health policy, Homelessness, Integrated systems, Health and housing

## Abstract

**Background:**

The ‘Everyone In’ national policy initiative launched in England during the COVID- 19 pandemic provided accommodation and health and care support to people who were (or at risk of) sleeping rough. This study aims to understand what worked well and less well in implementing ‘Everyone In’ for improving physical and mental health outcomes for people experiencing homelessness.

**Methods:**

Between January and October 2023, in-depth interviews/focus groups were conducted across England with those involved in the delivery/implementation of ‘Everyone In’ and those accommodated. Framework analysis and case study analysis were used for a contextual understanding of the implementation of the policy initiative.

**Results:**

Twenty-five people accommodated through ‘Everyone In’ (28–58 years; 88% males) and 43 service providers (25–62 years; 40% males) were interviewed. Flexibility in funding and resources, ‘joining up’ services/support, and innovative responsiveness in services across health, care, and housing systems were key positive features of the initiative. In the long term, ‘Everyone In’ has provided positive learnings for delivering holistic and integrated health and social care. It has also highlighted the importance of accommodating psychosocial needs and addressing the complexities of alcohol and substance use in all homelessness strategies.

**Conclusions:**

Pathways to care for people experiencing homelessness need to be flexible and responsive. Complexities such as substance use need to be approached with compassion while addressing the role of wider determinants in such health behaviours. Innovative approaches and joined-up work improve delivery of interventions and integrated care can reduce barriers to access to support.

**Supplementary Information:**

The online version contains supplementary material available at 10.1186/s12913-025-12713-w.

## Background

Homelessness is a significant and growing societal and public health challenge in the UK and globally. People experiencing homelessness, particularly those sleeping rough, experience an increased risk of adverse health outcomes. Global literature indicates that people experiencing homelessness have 8 to 12 times higher mortality than the general population, largely due to drug-related deaths, accidents, cardiovascular diseases, and infectious diseases [1]. People experiencing homelessness with alcohol or drug dependence are more than twice as likely to attend the emergency department in the UK [2]. In addition, they have higher rates of hospital admission compared to the general population for varied problems (e.g. skin conditions and long-term conditions such as epilepsy and angina) that might be preventable and treatable with better access to community-based health and care services [3]. With the launch of the Rough Sleeping Strategy in 2018, the UK government aimed to end rough sleeping [4]. However, the COVID- 19 pandemic presented new challenges for the government. This included: addressing isolation concerns for people experiencing homelessness when rough sleeping or living in temporary accommodations such as hostels; and their increased vulnerability to the virus and its consequences due to their underlying health conditions. In response, the UK government announced £3.2 million in emergency funding to all the local authorities (local government branches) in England to help provide accommodation to people who were (or at risk of) rough sleeping during the pandemic lockdown [5]. This policy initiative was called ‘Everyone In’. Subsequently, local authorities across England commissioned hotels and other accommodations such as bed and breakfasts, student housing, and holiday rental accommodation and worked with agencies across the health and care sector to provide food, health support and care as needed [6].

The rollout of the ‘Everyone In’ policy initiative presents a unique opportunity to learn from new ways of working and innovative approaches that were developed in offering accommodation along with delivering health and social care to those sleeping rough. There have been efforts to evaluate the impact of ‘Everyone In’, in addition to the several reports published on the impact of COVID- 19 on the homeless population [7–9]. These reports, included data collected during the pandemic (2020–21) and explored the impact of ‘Everyone In’ on homelessness while also reporting difficulty in access to health services, broadening provision to include migrant populations facing homelessness, and overall costs of providing emergency accommodation to people rough sleeping at the time of the pandemic. Examples of positive innovations in current evidence have included the delivery of opioid agonist treatment [10] and increased access to primary care [11].

However, a gap remains in understanding the wider physical and mental health impacts of the ‘Everyone In’ initiative on people experiencing homelessness. This qualitative study aimed to explore what aspects of ‘Everyone In’ worked well and less well for improving physical and mental health outcomes, including substance use. This study, which was conducted after ‘Everyone In’ ended, also explored long-term learnings that can be translated into future practice and policy.

## Methods

### Study design

A qualitative study was conducted in different towns and cities in four regions of England– North East, North West, South West and London.

### Study participants

In-person and telephone interviews were conducted with adults (aged 18 years and older) who self-identified as having been provided accommodation through the ‘Everyone In’ initiative (service users). They were recruited using a purposive recruitment strategy supplemented by snowball sampling across the four regions. Recruitment was facilitated through pre-existing networks of the homelessness charity partner involved in the study, Groundswell. The service users were contacted either via a social media post or directly approached during their visit to the homeless shelters or hostels. In either case, their eligibility was first established by asking about their living arrangements during the pandemic.

In addition, virtual interviews and in-person or virtual focus groups were conducted with policymakers and people involved in commissioning, planning, managing or delivering health and housing support during ‘Everyone In’ (service providers). Participants were purposively recruited based on location (from four sites), role (mix of practice, policy and commissioning), and organisations representing the health and care system (including local authorities, the voluntary sector and the National Health Service (NHS)). The identified service providers were then sent an email inviting them to participate in the interviews and in some cases followed up via telephone calls if needed.

### Data collection

A semi-structured topic guide was developed for each participant group based on input from stakeholders in local authorities, policy and people with lived experience of homelessness (copies of the preliminary topic guides have been included as *Supplementary Files*).

Interviews were conducted between January and October 2023 by trained qualitative researchers (NJ, KH, DB, SM) and were on an average 30 to 45 min in duration. All interviews with service providers were conducted online whereas those with people experiencing homelessness were either conducted online, via telephone or in person at homelessness shelters or hostels. In addition to the interviews, four focus group discussions were conducted between July and October 2023 by two researchers (NJ and EAA). Two focus group discussions were conducted in-person in Newcastle upon Tyne, which included 4 and 5 people respectively, and two were conducted online, each including 2 service providers. Participants within the focus groups were from the same organization and were involved in the delivery of the ‘Everyone In’ initiative, however, had some variations in their roles and hence were included in group discussions to gather understanding from different perspectives. The team met regularly to discuss the appropriateness and refinement of questions, progress in recruitment, and initial reflections on the data. All interviews/discussions were conducted in English and audio recorded with consent. Service users received a £25 voucher for a grocery store as a thank-you for participating. Recruitment continued until data sufficiency had been reached and no new themes were identified [12].

### Data analysis

All the data were transcribed verbatim and then anonymised before being stored in password protected folders on a secure university server, accessible only to the research team. Transcribed data were organised, coded, and accessed using NVivo software v.14 [13]. An iterative process was used to collect data, which was then analysed using a framework approach [14] wherein after coding the first few transcripts by two researchers (NJ and KH), an analytical framework was defined to code the remaining transcripts and develop initial themes and sub-themes. Final themes were developed by considering data across transcripts from both service providers and service users and identifying common themes from across the data. Where the perspective of one participant group was stronger than the other, this was noted in the results. Further, a case study analysis [15] was conducted to provide an in-depth and contextual understanding of specific examples to identify shared learning and good practice for future policy and practice. Any instances of innovations in implementation or of challenges were treated as cases and explored through the lens of different participants (both service users and providers). Throughout the results, illustrative quotes have been presented and participants have been identified based on their broader geographical region and participant group to maintain anonymity.

### Lived experience involvement and engagement

This study was undertaken in collaboration with Groundswell, a national homelessness charity and champion of involving people with lived experience in developing solutions for homelessness. Groundswell facilitated input into this study from over 10 people who had experienced homelessness. This took place in the form of workshops conducted across the study stages to gather input on various elements of the study including ethics application, recruitment strategies, topic guide development, initial themes and dissemination plans. There were opportunities for people to feedback at multiple workshops at different time points or offer input on a one-off basis.

### Ethics

Ethical approval was obtained from the Faculty of Medical Sciences Research Ethics Committee, part of Newcastle University’s Research Ethics Committee (Ref: 21140/2022). Additionally, approval was obtained from the Health Research Authority and Health and Care Research Wales (Ref: 22/HRA/4834) for the involvement of NHS staff.

## Results

Interviews were conducted with 25 service users. Participants reported that they had been provided accommodation in hotels, hostels, apartments and student housing. Interviews or focus group discussions were also conducted with 45 people involved in the delivery or implementation of the initiative. Demographic details of the study participants are provided in Table 1 and have been aggregated to maintain anonymity.

**Table 1:** Demographic characteristics of the study sample (*n*= 70)

**Service users (SU): People provided housing through ‘Everyone In’ (*n*** **= 25)**		
**Age range**		28–58 years
**Gender, N (%)**	Male	22 (88%)
	Female	3 (12%)
**Ethnicity, N (%)**	Black African, Black Caribbean, or Black British	11 (44%)
	White British	10 (40%)
	Other ethnicity	4 (16%)
**Location, N (%)**	London	9 (36%)
	North West	7 (28%)
	North East	6 (24%)
	South West	3 (12%)
**Service providers (SP): People involved in delivery or implementation of ‘Everyone In’ (*n* = 45)**		
**Age range**		25–62 years
**Gender, N (%)**	Male	18 (40%)
	Female	27 (60%)
**Ethnicity, N (%)**	White British	42 (93.3%)
	Other Ethnicity	3 (6.7%)
**Location, N (%)**	North East	15 (33.3%)
	North West	11 (24.4%)
	South West	10 (22.2%)
	London	9 (20%)

There were three major themes and 3 case studies which illustrate what worked well and less well in terms of the provision of health support, partnership working and relationship building, and legacy of ‘Everyone In’.

### Responsive health support

#### Adaptability in service provision

Service providers reported that, due to the pandemic and the ‘Everyone In’ initiative, the way services were provided to service users changed to become more flexible and innovative. Despite the uncertainties and the need to respond urgently, the situation quickly transformed into one where several partners and teams came together to set up different hotels/accommodation. It was noted that increased flexibility allowed for housing and healthcare providers to adapt, experiment and respond in new ways and a lack of ‘red tape’ allowed services to be implemented quickly and to improve accessibility for service users. For instance, a service provider from London explained that once they got people accommodated, they were quickly able to “*change their prescription to the local pharmacy”* (SP1, London)*,* which was near the hotel where they were accommodated and arrange for the clients to reach the pharmacy.

*“Everyone In allowed us to work in a completely different way to how we had before. We had flexibility that we've never had before. And all of a sudden, a lot of the governance and red tape had to be pushed to one side.”* (SP2, North East)

It was reported that when service users and health and social care providers come together at the same location (in this case the hotel), previous challenges related to access (such as multiple appointments or travel) were overcome.

*“…we were pretty much full-time located at the hotel now… Actually, in a weird sense, it was an opportunity to work with the people who we wouldn’t often be able to engage. So, we saw it as an opportunity.”* (SP1, London)

*“They were actually there on your doorstep rather than you having to make an appointment and go and see them.” (*SU2, South West)

However, some of the challenges in providing health support at that time came with online or telephone consultations or limited digital access. This meant that service users needed access to mobile phones and credit.

*“It was totally different in that this time you couldn’t get to see the people that you wanted to see. Everything was just appointments over the phone. Say if you had no credit on your phone, you were stuck really.”* (SU3, North East)

Service provision in some local authorities expanded to include those with no recourse to public funds, and ethnic minorities, which was a first in many cases. A case study illustrates this in Table 2.

**Table 2:** Case study exploring accommodation and provision of health support inclusive of people with no recourse to public funds

‘Everyone In’ offered accommodation to people with no recourse to public funds. This practice, which was not common before due to statutory limitations, was now possible due to the broader inclusion mandate of the initiative. This allowed service providers to reach out to those people who were previously excluded from homelessness strategies: *“…This cohort started to hit the street…and rough sleepers were entitled to hotel accommodation, so we took full advantage of that fact and got them in… there was people from all over the world, but there was a lot of Eastern European guys who had obviously been working in the informal economy for quite a long time, but they just had no rights, they basically had just been kicked out of wherever they were staying, literally for being symptomatic.”* (SP2, London) To address challenges in providing health support to people from non-English speaking backgrounds, providers from London engaged with translators to bridge the gap in communication. One explained: *“We used our telephone translation account, and we just took the number wherever we went, and we made sure our staff had work phones so that we could use telephone translation services.”* (SP5, London) Other than the language barriers, certain population specific behavioural factors also had to be considered, such as the use of specific kinds of substances in different boroughs of London: *“it’s interesting how homelessness in different boroughs presents in different ways. And drug use is different so like where Westminster might be all crack* [cocaine] *and heroin, you go to Ealing, and it’s loads of alcohol and maybe like chewing tobacco and other kinds of drugs that perhaps we don’t encounter that much in Westminster.”* (SP6, London) Some regional service providers have also reported that they continue to provide support to people with no recourse to public funds and “not look further into people’s circumstances”, at the back of the ‘Everyone In’ initiative	

#### Unintended health consequences from being accommodated

For some service users, having individual rooms (instead of shared accommodation) resulted in unintended consequences in terms of isolation. Service providers reported that people with substance use issues and mental illness found it difficult to stay indoors.

*“But now I’m in hotel, with the TV on and I’m feeling very lonely. I’m not saying the homeless people were a part of the family, but they were still on me with this journey, and there was a sense of togetherness.”* (SU7, North West)

*“I think mental health was the issue of being isolated, being scared, being quite vulnerable. A lot of people that we brought in had not been off the streets for a long time, years, and to come in and be in a hotel room, it was quite difficult for them.”* (SP1, London)

Lack of access or availability of alcohol or other substances and the “*no drinking rule”* (SU6, London) in accommodations led to some people reporting serious withdrawal symptoms*.* Cases of violence were also reported which eventually led to evictions from the accommodation, thereby precluding access to health support within the accommodation and creating a discontinuity in the care.

*“I got arrested three times while I was in there. I had some vodka, and it came out sideways* [angered him up] *… and that I started going at them* [other people] *then through the drink. I got arrested and beat up by the police, pepper sprayed within an inch of my life… And then third time they kicked me out”* (SU2, South West)

Table 3 below illustrates a case study which elaborates on examples of unintended consequences of the ‘Everyone In’ initiative.

**Table 3:** Case study examining accommodation environment and mental health of service users

Some service users faced challenges with their mental health. They found it difficult to stay indoors, especially when sufficient support for mental health and substance use was unavailable. While this was not universal, some people reported feeling “*isolated*”. Constant fights broke out in their accommodations and many people were eventually evicted. Service providers also reported having to buy alcohol to reduce withdrawal symptoms in the service users with alcohol dependence to offer harm reduction strategies *“The charity had a no drinking rule… you've just put a bunch of alcoholics in to a hotel and then tell them not to drink… It's seriously not understanding addiction…I'd say in the 48 h I was there at least a third that were taken in were back out on the streets.”* (SU6, London) Service providers reported that due to the lack of resources at the time of the pandemic, mental health providers were sometimes redeployed, leaving an unmet need for service users *“…At a time when the entire population’s mental health was struggling because of social isolation and everything, it was a very scary time and they were taken out of the homeless sector and they were remobilised into, redeployed into a different job* [due to low priority]*…”* (SP5, London) This was echoed by those who were accommodated who felt the changes to service provision left gaps: *“She was as helpful as she could be within her remit… She wanted me to go to council offices, but I didn’t want to go to the council office to explain why my mental health was going down. I wanted her to come to me, but she wouldn’t. No one came*.” (SU7, London) Furthermore, while pharmacies continued to be open during the pandemic, travelling to them daily for collecting opioid agonist treatment was often a challenge for service users. As a result, in some cases, pharmacies would have to dispense more than one day’s script at a time, which in some cases was not viewed positively: *“When I first got out, they were having to give people fourteen days* [of methadone] *to take away with them. Obviously, if people are normally going once a day to pick it up, getting fourteen days’ worth of methadone to take away with them people were lapsing…it was just making things worse.”* (SU3, North East) On the other hand, some service users, especially people who use alcohol, reported that the environment of the accommodation provided motivation to *“changing their lives for the better”.* Service users reported that the service providers would arrange for group sessions in common spaces of the hotel to encourage people to talk about their struggles. Talking about the peer support in the accommodation, someone said: *“We were sitting around, just seven, eight people, recovering alcoholics. That was a big boost for me. I was seeing a stranger just opening up and really devoted to leaving alcohol alone. That just made me push through.”* (SU1, South West)	

### Partnership working and relationship building

#### Relationships between service providers and service users

Trusted relationships between service users and providers were perceived to increase access to health and social care support. Being linked with support workers was viewed positively by service users since the support workers helped by acting as a point of support or “liaison” for engaging with the health services.

*“I think there were certain things that worked well, the way that the commissioned services staff were with people and the relationships that they had in helping them to do things through those relationships and helping them to understand things through those relationships, helping them to isolate or get what they needed”* (SP3, South West)

Speaking about support workers, one person explained, “*He gave me new confidence in myself. Assertive. Built up my confidence. From there, every day it was a little bit. Now, what I am now, I feel much better.”* (SU3, London).

#### Inter-sectoral relationships

Many services reported that one of the things that worked well during ‘Everyone In’ was the joined-up working across sectors/services. Service providers reported partnerships and cross-sector working allowed them to break barriers in accessibility by bringing services together. Different sectors were often co-located in the same hotel or region together (for example, housing, health, welfare, mental health, and vaccination), and were supporting each other through sharing of resources and knowledge which made implementation of health services, physically (i.e., in same location) and strategically, easier than before.

*“There was a lot of just coming together and firefighting and brainstorming and thinking on your feet very quickly. But as the time progressed, I think there was more and more services came in, again, mental health, substance misuse services, physical health services, nursing*.” (SP1, London)

While many partnerships were reported to be pre-existing, ‘Everyone In’ gave a chance for these partnerships to be strengthened due to joined-up working and constant communication with the common goal of providing health and social care support to people experiencing homelessness during the pandemic.

*“So that became a thing, and increased connectivity with housing and homelessness providers… I’ve had far more conversations with Public Health since then, you know, we weren’t very involved with them before.”* (SP5, South West England)

Partnership working allowed services to be delivered quickly, which was also noticed by service users, as one person said, *“It took them…not even weeks, where the time before that they took about eight months [laughter] to sort it out.”* (SU2, North East) In some cases, this happened due to easy data sharing between agencies. However, in some cases refusal to share data within the partnerships also led to a delay in delivery of support.

*“It was sometimes literally on the ground at a hotel with a whole team mobilised there for the day who’ve been told we’re not allowed to make calls to people because they won’t disclose any of that.”* (SP6, London*)*

Table 4 below illustrates how partnership working enabled improved implementation of health support to service users.

**Table 4:** Case study with example of services coming together and providing wrap around support for service users

The ‘Everyone In’ initiative helped improve access and engagement to health services through joined-up working and coordination with health and care providers and outreach into accommodations for service users. This case study elaborates on examples of wrap-around support from services in South West England. One key frontline worker from a voluntary sector organization explained: “*We were working with the city council identifying people. We would then be doing a sort of triage of whether we felt that they in the hotel would be a good match…based on their needs, safety, location of the hotel. We were working alongside the hotel staff, and some security staff… It was something that had not been done before*.” (SP4, South West) After being triaged and provided a suitable accommodation, people were linked with a support worker: *“Everybody that came into the hotel was allocated a key worker. The number of people that any individual case worker was working with wasn’t too high… There was a real focus on mental health but also on the ability to manage, so we worked closely with adult social care, physical health services, drug, and alcohol services. We were really looking at a holistic approach.”* (SP4, South West) Service providers overcame challenges in access to mental health services by providing video recorded messages to service users. This also allowed for provision of mental health support while maintaining social distancing requirements *“…It was led by a consultant psychologist within drug and alcohol services…they created a series of video modules that were about 30–40 min, each one. Initially, it was created to prepare people for detox, but it was a good psycho-education tool for people who were struggling with their mental health based on traumatic experience. We were able to use that with people and sit with clients in the hotel and watch a video, talk through it…”* (SP4, South West) In addition to working with a range of partners to provide health and care support to the service users, they also worked together to create a safe and non-stigmatising environment for the people with lived experience of homelessness by training hotel staff and security teams: *“We did some training for the three teams together around safer spaces and psychologically informed spaces…We had a clinical psychologist as part of the team and we were able to utilise her resource to come and provide regular reflective practice for, the support staff, the hotel and security staff as well…”* (SP4, South West) These positive efforts were also felt by service users. One person explained: *“They offered a group consultation… But after just being able to talk with them, they encouraged us to communicate with each other. And every evening, just sit around and talk about ourselves. It felt like the world was just dawning on me, and I’m grateful.”* (SU1, South West)	

### Legacy of ‘Everyone In’

#### Reinforced funding and support for health and homelessness

Service providers across different local authorities reported receiving various streams of support after ‘Everyone In’ to improve homelessness strategies informed by the learnings from the pandemic, while also considering health outcomes of service users. These funding streams, which were provided off the “back of Everyone In’’ (SP2, North East), have encouraged flexible provision models in some cases, however, some providers have also reported the funding streams to be “short-term” (SP5 and 6, London; SP7, North East; SP2, North East).

*“We have got money and we’ve used that money to create 125 dedicated move-in flats that we call Ready to Live and that has helped so far, I think it might change soon, we’ve managed to avoid putting people into bed and breakfasts and accommodation that isn’t designed to live.”* (SP6, North East)

Flexible provision models allowed some regions to extend some of the provisions for people with no recourse to public funds to continue providing them with support, with a study participant reporting that *“now we do far more on that front.”* (SP7, London).

In terms of changes in support, service providers reported that learnings from ‘Everyone In’ led to the introduction of more outreach support for issues related to substance use.

*“…It prompted more outreach, which has been ongoing. And I think offering rapid access to opiate substitutes helps people engage.”* (SP5, South West)

#### Innovations in working

The ‘Everyone In’ initiative encouraged different sectors to introduce changes in the way housing or health services are being provided to service users. Some service providers reported that current strategies or programmes for homelessness consider inclusive health support and that they are moving away from shared accommodation to single occupancy rooms to provide more ‘dignity’ to the service users.

*“I think it had an impact... what would accommodation look like in the future rather than having lots of shared spaces. Not just for the health reasons, for the mental health reason and for being dignified reason. And some organisations went away from delivering a night shelter to delivering activities, programmes instead… And it gave a really good opportunity for co-production as well in making these new services.”* (SP10, North West)

In terms of partnerships, the experiences of service providers differed regionally. While some service providers reported thinking that the way organizations work together has now changed because of the experiences of ‘Everyone In’, others think that while the partners remain on the horizon, the partnerships have not been the same.

*“I think definitely a whole bunch of people came together in a new way, but it’s fizzled out a bit, really.”* (SP5, South West)

Service users had limited experience of any changes in the type of support received after ‘Everyone In’ ended. Some study participants felt that services have gone back to the usual way of working.

*“They have learned quite a bit from it but they’re starting to take a bit of a step back and slipping back into old habits…?”* (SU3, North East)

In contrast, others looked back at ‘Everyone In’ positively and reported that the health support received during the pandemic was helping them three years on.

*“Sometimes when I look at alcohol, I’m just thinking about what they told us, “Just one more day, just one more day,” you know. And pushing, pushing.”* (SU1, South West)

## Discussion

‘Everyone In’ was the first national policy initiative in England (launched during the COVID- 19 pandemic) which attempted to coordinate access to accommodation and delivery of health and social care for people who were, or at risk of, rough sleeping. This study found that ‘Everyone In’ implemented a flexible model of service provision that integrated health with housing and social care. It found that dedicated funding, flexibility in service provision strengthened relationships and partnerships as well as ‘joined-up’ services. It also led to improved engagement with health services by service users. In the long term, ‘Everyone In’ has provided positive learnings for delivering holistic and integrated health and social care. It has also highlighted the importance of accommodating psychosocial needs and addressing the complexities of alcohol and substance use of people in all homelessness strategies.

Other studies published during the pandemic have found that flexibility in the delivery of interventions is crucial in improving access to health services by people experiencing homelessness [16]. Similar findings were found in our study wherein flexibility and responsiveness across the system (including housing, health, voluntary sector, and NHS) supported the provision of coordinated housing and health care for people currently or at risk of sleeping rough. Harm reduction strategies, such as buying alcohol for service users, indicate the complexities involved in solving homelessness and highlight the importance of flexibility in approach, which often requires coordination across sectors. Individual experiences published elsewhere, also during the pandemic, report that such harm reduction strategies when implemented in a controlled environment with secure housing (during ‘Everyone In’) have helped people experiencing homelessness address their alcohol and substance use [16, 17]. Guidelines surrounding opioid agonist treatment were also an interesting practice during the pandemic, wherein multiple days’ worth of prescription was given to service users, which led to fewer visits and reduced the perception of stigma by service users. While in our study, some participants reported that this led to issues surrounding the monitoring of doses, a report published by a homelessness charity organization showed that people experiencing homelessness and living with substance use issues preferred getting weekly or fortnightly methadone instead of daily and this increased their trust in general practitioners (GPs) [18].

Another significant learning was the importance of partnerships and joined-up working between organisations and different sectors within health, housing and voluntary sectors, a finding echoing prior research conducted in 2019 and 2023 respectively in this area [19, 20]. This approach was helped by pre-existing strong relationships between these sectors, while in other places partnership working led to innovation in how services/support was provided. These partnerships are particularly pertinent in the context of Integrated Care Systems, which bring together health, care, voluntary sector and Local Authorities [21]. Although service user perspectives were limited, joined-up working was seen as a positive by some of the service users, particularly among those who were readily able to engage with support, as well as by different service providers in terms of having a mutually supportive environment. It may be worth noting that ‘Everyone In’ has provided a good example of a responsive policy which utilised evidence-based strategies explored previously in the literature. For example, other studies conducted during the pandemic have reported physical distance between services to be a barrier to access for people experiencing homelessness [22]. Our findings highlight that co-located interventions that address multiple needs remove some of these barriers and present learning for outreach interventions, especially where privacy of the service users is not a challenge. Other studies by the co-authors (2024) that have looked at health and health services in people experiencing homelessness have found that integrated health and housing can be useful in improving outcomes related to substance use [23]. Prioritizing funding, integrated care and good relationships between service providers and people experiencing homelessness have also been reported to help in improving health interventions [24].

Important legacies of ‘Everyone In’ have been seen in different regions. While the government has reiterated that the law on no recourse to public funds hasn’t changed, some councils were able to temporarily ease some restrictions for citizens from European Economic Area (EEA) to provide accommodation and support [25]. The positive learnings from ‘Everyone In’ were also reflected in national guidelines released thereafter. For instance, the National Institute for Health and Care Excellence (NICE) released guidelines for integrated health and social care for people experiencing homelessness in 2022 [26] These guidelines reflected the importance of coordinated care (between health, housing and social care) to improve engagement with health and social care by people experiencing homelessness. It emphasized the importance of appropriate funding and multidisciplinary teams to improve care provision.

### Strengths and limitations

A strength of the study was the ongoing and iterative insights from people with lived experience of homelessness. This helped us refine the language used to clearly identify service users and framing of questions in the topic guides. Additionally, the iterative process enabled us to adapt approaches to identify new organisations and networks for recruitment based on ongoing feedback when there were challenges identifying potential participants. Another strength of the study was the breadth of insights gathered from key stakeholders including policy, healthcare providers, local authorities, voluntary sector, peer workers, commissioning and people with lived experience. Our study was limited by the fact that the interviews and focus group discussions happened nearly three years after ‘Everyone In’ was first implemented. In terms of gathering information from services and commissioners, this sometimes meant that relevant people had moved on from the roles they had during the pandemic. The research team took time and followed connections to identify the right people to gather information. In some cases, people found it difficult to recall information. However, in most cases, it gave people a chance to reflect on the longer-term learnings and legacy of ‘Everyone In’. The issue of timing was more of a challenge in terms of identifying service users, who had been accommodated during ‘Everyone In’. Often people who took part in ‘Everyone In’ had moved on or were unclear if their housing had been provided specifically through this policy initiative. We tried to overcome this challenge by modifying our recruitment strategies in consultation with people with lived experience of homelessness. Additionally, Groundswell, who led the data gathering from service users, were able to reach out to wider networks to engage with people who were part of the ‘Everyone In’ initiative. In terms of numbers, our study had a greater number of service providers as compared to service users as our study participants. This risked some of our themes to be more defined by the data from service providers, however, we attempted to analyse the data from the perspective of both participant groups. While most themes and sub-themes included data from both service providers and users, long-term legacy of ‘Everyone In’ was discussed less by service users. Another limitation is that our study did not explore comparisons and differences in the policy roll out across the different geographical sites. Nonetheless, the results draw out findings that were pertinent across the different sites following the national roll-out of the policy.

### Implications and conclusions

Our study begins to address the knowledge gap on the role of policy in bringing together housing, health and care by providing the learnings from a responsive nationallevel initiative in England that offers important insights for policy and practice. The findings point to the potential for change that can happen at speed and scale following policy shifts. In this case, a change in national policy resulted in closer working of services in health and non-health arenas. Innovative approaches and joined up working across multiple agencies in service delivery have the potential to reduce barriers to access to support. Our findings show the benefits this coordinated or integrated way of working can have on improving engagement with health services for populations who otherwise poorly engage with services. Another key implication for practice is the benefits of adaptability, flexibility and responsiveness in service provision and partnerships between organisations and sectors, which can help overcome challenges in access, finding relevant information, and navigating services. Improving physical and mental health of people experiencing homelessness require consideration of outreach support and co-located services. The engagement of trusted peers or support workers can act as a bridge between services and people. Additionally, complexities such as substance use need to be approached with compassion while also addressing the role of wider determinants (such as housing, and welfare) in the prevalence of these health behaviours.

## Supplementary Information

Supplementary Material 1.

Supplementary Material 2.

## Data Availability

The data generated and/or analysed during the current study are not publicly available due to the highly sensitive nature of the data and to protect participant’s confidentiality as they could contain potentially identifiable information, but summaries are available from the corresponding author on reasonable request.
