# Supplementary material for: Learnings from providing integrated health, housing and wider care for people rough sleeping during the COVID- 19 pandemic: a national qualitative study of the ‘Everyone In’ policy initiative
Source: BMC Health Serv Res. 2025 Apr 15;25:549. doi: 10.1186/s12913-025-12713-w (PMC11998433; doi:10.1186/s12913-025-12713-w)
Supplement: Supplementary file 1 — Supplementary Material 1. [file 12913_2025_12713_MOESM1_ESM.docx]

**Topic Guide – for interviews with people experiencing homelessness who were provided accommodation through ‘Everyone In’**

**Title: Learnings from providing integrated health, housing and wider care for people rough sleeping during the COVID-19 pandemic: a national qualitative study of the ‘Everyone In’ policy initiative**

1. Thinking back to the beginning of 2020 (just before the lockdown), can you tell us about your living situation at that time?
   1. Where were you living? Were you in that situation for a long time?
   2. Were you living on your own or with other people – this could be your partner or friends or any family?
2. Can you tell me about how your housing changed during 2020 (during the lockdown)?
   1. Where did you live during the lockdown? How long did you live there?
3. I was hoping to ask you a little bit about your health before the lockdown –
   1. What was your health like then? Any concerns about your physical health (such as difficulties in breathing), mental health or substance use?
4. Thanks for sharing, we want to understand a bit more about what support or help you had with your health during the time you were provided with accommodation during the lockdown – especially, if there was any different kind of support you received. This could be to do with physical health, mental health or substances or all of these issues.

- 1. What exactly did you get in terms of support for your health? Who provided it (e.g., support workers, etc)?
  2. Was this different to the support you had for your health before that time? In what way was it different? Did you get any extra help or support?
  3. Did anything make it easier or harder for you to get the support you needed? What helped?

1. Following on from the support you talked about, did you notice different teams/ support workers coming together to help you get the help you needed for your health? This could be something like support workers coming to the accommodation to talk about your mental health or getting help with medications or prescriptions.
   1. Did the different services work well together or maybe not so well?
   2. Did you feel like this helped with your health issues?
   3. How did you feel about these services connecting or coming together? Were you comfortable with that?
2. So, two years on, has all this support you had then helped with your health?
   1. Have you continued to get that help/ support for your health in the same way? Is it easier or harder now to get help? Why?
3. Is there anything you would like to say that has not been covered so far?
