# Supplementary material for: Learnings from providing integrated health, housing and wider care for people rough sleeping during the COVID- 19 pandemic: a national qualitative study of the ‘Everyone In’ policy initiative
Source: BMC Health Serv Res. 2025 Apr 15;25:549. doi: 10.1186/s12913-025-12713-w (PMC11998433; doi:10.1186/s12913-025-12713-w)
Supplement: Supplementary file 2 — Supplementary Material 2. [file 12913_2025_12713_MOESM2_ESM.docx]

**Topic Guide – Interviews (with Service Providers - including frontline staff, LA leads, and commissioners)**

**Title: *Learnings from providing integrated health, housing and wider care for people rough sleeping during the COVID-19 pandemic: a national qualitative study of the ‘Everyone In’ policy initiative***

1. We want to start by understanding your involvement with ‘Everyone In’. Can you tell us about your role in the ‘Everyone In’ initiative?
   1. How long were you in this role?
2. What did ‘Everyone In’ look like in your area?
   1. What types of services were provided under ‘Everyone In’?
   2. Who provided these services and what agencies were involved?
   3. Did these services include anything for the health and wellbeing of those individuals who were provided accommodation through ‘Everyone In’? Who offered these services? **OR**

Can you tell us more about the health and wellbeing support you mentioned, that was provided to those who were accommodated through ‘Everyone In’? Who offered these services?

1. Thinking back to the support around health during ‘Everyone In’, what aspects of ‘Everyone In’ worked well particularly in delivering that health support when integrated with housing?
   1. What was behind that or led to or drove that good practice?
2. And was there anything you think worked less well in delivering health support to those who were provided accommodation through ‘Everyone In’?
   1. Were there any challenges in delivering that support for health-related issues to those provided accommodation through ‘Everyone In’?
   2. Did anything help with overcoming those challenges?
3. People experiencing homelessness often have barriers engaging with health services – so were there any aspects of ‘Everyone In’ that helped them overcome those barriers and engage better with health services?
4. By contrast, were there any barriers or unintended consequences faced by individuals while trying to engage with health services during ‘Everyone In’?
5. A key feature of ‘Everyone In’ is that different organisations across the local systems had to work together to deliver better health for people sleeping rough – could you tell me how those different organisations across the system came together in your area?
   1. Who were the different organisations or services that came together to provide the services?
   2. Were these new partnerships or pre-existing ones?
   3. What factors encouraged these partnerships to form across the system?
   4. Would it have helped having any other services/ organisations, who were not in this partnership? What services were these?
   5. Were there any challenges in all these different organisations across the local system coming together?
6. Did the organisations coming together help or have positive (or negative) impacts on health or services at that time – this could be related to the physical, mental health, substance use of those provided accommodation through ‘Everyone In’?
7. Did you find ‘Everyone In’ changed the way different organizations work together to provide wrap-around health care for people experiencing homelessness?
   1. You mentioned some partnerships earlier, have these continued even after ‘Everyone In’ ended? Have there been any positive impacts on wider partnership/collaborative working across the system? Why/ why not?
   2. Has there been any difference or impact on health of those experiencing homelessness as a result of these new collaborations and partnerships that were formed?
8. What is it like now for people experiencing homelessness to engage with services? Has it got better or worse? Are there fewer barriers now?
   1. Has ‘Everyone In’ played a role in that?
9. Are there any other lessons or learnings more generally for future health initiatives/policies around homelessness or rough sleeping?
10. Is there anything else that would like to add that has not been covered?
